# Enhancing Mental Health Through Retirement Planning Achievement: A Moderated Mediation Model and Income Group Differences

**DOI:** 10.3390/bs15111593

**Published:** 2025-11-20

**Authors:** Jing Yuan, Pengfei Jian, Buxin Han

**Affiliations:** 1 State Key Laboratory of Cognitive Science and Mental Health, Institute of Psychology, Chinese Academy of Sciences, Beijing 100101, China; yuanj09@yeah.net (J.Y.); jianpf@psych.ac.cn (P.J.); 2 Department of Psychology, University of Chinese Academy of Sciences, Beijing 100049, China

**Keywords:** retirement planning achievement, mental health, active social participation, retirement adjustment, household income

## Abstract

This study centers on retirement planning achievement, examining its impact mechanism on older adults’ mental health and its boundary conditions. Drawing on self-determination theory (SDT) and conservation of resources (COR) theory, we tested a parallel mediation and a moderated mediation model using data from an online survey with 900 Chinese retirees aged 55–74. Structural equation modelling revealed that retirement planning achievement directly and positively predicted mental health, and indirectly through three pathways: greater active social participation, higher retirement enjoyment, and reduced retirement loss. Furthermore, retirement adjustment exhibited dual, and opposing, moderating effects on the direct path: low retirement loss, as a psychological resource, significantly amplified the positive impact of planning achievement (a resource gain spiral), whereas high retirement enjoyment attenuated its effect (a ceiling effect). Income-group analysis revealed that both the parallel mediation and moderated mediation models were fully supported in the average-income group, but effects were non-significant for the insufficient-income group and weakened in the sufficient-income group. These findings suggest that retirement planning achievement represents a key pathway to promoting mental health in later life, but its benefits are constrained by psychological resources and socioeconomic status. The “achievement dividend” is greatest among well-adjusted retirees in the average-income group, highlighting the heterogeneity in retirement adjustment and providing evidence for targeted, equitable ageing-support policies.

## 1. Introduction

With the accelerating global trend of population aging, safeguarding and enhancing the quality of life and mental health of older adults has become a critical societal priority (World Health Organization, 2021). By the end of 2023, China’s population aged 60 and above had reached 297 million, accounting for 21.1% of the total (National Bureau of Statistics of China, 2024), presenting profound challenges to the pension system and eldercare services. Retirement, as a major life-course transition, represents both an opportunity to embark on a new chapter and a challenge in adaptive adjustment. The academic consensus holds that proactive retirement planning is a key driver of successful adaptation (J. H. Noone et al., 2009, 2010). Yet, a theoretical gap remains unaddressed. Prevailing perspectives—whether rooted in activity theory or continuity theory—tend to assume that maintaining post-retirement engagement naturally leads to later-life well-being. While such assumptions may be valid, they often overlook a more fundamental, antecedent question: to what extent are individuals’ pre-retirement plans actually realized? The transformation from intention to action to outcome is far from guaranteed, and this uncertainty constitutes a significant gap in the literature.

### 1.1. Theoretical Integration and Research Framework

To address this gap, the present study makes a clear distinction between retirement planning behavior and retirement planning achievement. We argue that achievement—defined as the extent to which planned goals are implemented and attained—may serve as the true bridge between prospective effort and positive retirement outcomes. At a deeper level, the achievement of plans represents more than goal attainment in the sense of goal-setting theory (Locke & Latham, 1990); it is the fulfillment of a “psychological contract” with one’s future self (Rousseau, 1995), carrying a psychological impact that is often more profound than the act of planning itself. This raises a central set of questions: through what mechanisms does planning achievement enhance mental health, and under what conditions is this mechanism most effective?

To answer these questions, this study integrates self-determination theory (SDT) and conservation of resources theory (COR) into a unified analytic framework. The two theories offer complementary explanatory power. Specifically, SDT elucidates why planning achievement can improve mental health—by satisfying older adults’ basic psychological needs in retirement: autonomy (living life on one’s own terms), competence (feeling capable and accomplished), and relatedness (maintaining connections with others) (Deci & Ryan, 2000). However, SDT alone does not fully address differences in the efficiency of this well-being conversion—namely, why some individuals benefit more from planning achievement than others. Here, COR theory fills the gap by explaining how existing psychological resources influence the conversion process. COR posits that individuals have an inherent tendency to protect, maintain, and build resources (Hobfoll, 1989). In this view, pre-existing psychological resources—such as a positive retirement adjustment state—can act as catalysts or buffers, moderating (amplifying or attenuating) the benefits of planning achievement as new resource input, thereby producing a “resource gain spiral” (Hobfoll, 2001).

By integrating SDT and COR, this study constructs a comprehensive framework to uncover both the internal psychological pathway (mediation) through which retirement planning achievement influences mental health and the boundary conditions (moderation) that determine its effectiveness. This approach allows us to answer the core question of “for whom and how planning brings well-being” more systematically. Grounded in this framework, we test a moderated mediation model and further examine its variations across different income groups, seeking to provide both theoretical insights and practical implications for fostering adaptive and fulfilling retirement among older adults.

### 1.2. Retirement Planning and Retirement Planning Achievement

Pre-retirement planning is a goal-oriented behavior through which individuals prepare for their post-work life. It helps individuals establish realistic expectations about the potential changes during the retirement transition (Peeters & Van Emmerik, 2008) and set clear long-term goals for life after retirement (Topa et al., 2009). This forward-looking preparation is crucial not only for the quality of life and well-being of retirees but also for alleviating societal social security pressures (L. Y. Zhang & Wang, 2019). Empirical research consistently demonstrates that individuals who plan for retirement tend to adapt better and report higher well-being. Both cross-sectional and longitudinal studies have found that more comprehensive pre-retirement planning is associated with multiple positive outcomes, including better physical and mental health (M. Wang, 2007; Yeung, 2013), more positive retirement attitudes and adaptability (Reitzes & Mutran, 2004; Muratore & Earl, 2015), and higher life satisfaction (Topa et al., 2009; J. Noone et al., 2013). Building on the resource dynamics model (M. Wang et al., 2011), Yeung and Zhou (2017) further revealed the underlying mechanism between planning activities and post-retirement well-being. They found that retirees who engaged in more pre-retirement preparation acquired more resources initially, which contributed to positive changes in their well-being over time, confirming that pre-retirement planning effectively predicts post-retirement quality of life. However, these studies harbor a theoretical leap: they often assume that planning intentions or behaviors directly translate into positive outcomes.

To address this gap, the present study makes a crucial distinction. Retirement planning achievement, which refers to the process of putting specific plans into action and attaining the desired effects, represents the final stage of retirement decision-making (Feldman & Beehr, 2011). The threshold for achieving retirement plans is higher than that for merely executing them, and achieving plans is more challenging. Plans that are executed but not achieved may fail to enhance the security and well-being of older adults in later life (Wu & Chao, 2024). Building on the finding that retirees’ well-being improves with increased retirement resources (Yeung & Zhou, 2017), we argue that planning achievement, rather than planning behavior alone, serves as the crucial driver or primary conduit linking prospective effort to positive retirement outcomes. This study does not negate the importance of planning behavior; rather, it aims to deeply analyze the downstream phase of this mechanism—the process of achievement and its impact on mental health—to deepen our understanding of the planning-well-being relationship. Indeed, as Wu and Chao (2024) noted, although both executing and achieving plans are beneficial during retirement, retirement planning achievement not only has a positive impact but also provides a greater boost to older adults’ well-being, serving as the actual bridge between prospective intentions and positive adaptive outcomes.

From a theoretical perspective, retirement planning can be viewed as an informal psychological contract with one’s future self (Rousseau, 1995), encapsulating personal expectations and commitments. Achievement represents the fulfillment of this contract—an application of goal attainment theory to the retirement domain (Locke & Latham, 1990; Emmons, 1986). Successful fulfillment, transforming a blueprint into reality through personal effort, directly affirms an individual’s autonomy and competence, fostering a strong sense of control and self-efficacy (Bandura, 1997). Conversely, failure to fulfill such plans constitutes a perceived contract breach, which may elicit negative effects that could even outweigh those of having never planned at all (Festinger, 1957).

The core argument of this study is that the process of planning achievement enhances mental health by satisfying basic psychological needs. Self-Determination Theory (SDT) offers a perfect theoretical framework for this proposition (Ryan & Deci, 2000). According to SDT, human well-being and intrinsic motivation stem from the universal satisfaction of three basic psychological needs: autonomy (the experience of one’s behavior as self-chosen and volitional), competence (the feeling of being effective in dealing with environmental challenges), and relatedness (the feeling of being cared for and connected to others). The successful achievement of retirement plans is a prime manifestation of these three needs being met. First, autonomously developing and executing a retirement plan is an exercise of personal choice, directly satisfying the need for autonomy. Second, transforming a blueprint into reality, overcoming potential difficulties along the way, provides a strong sense of efficacy and accomplishment, satisfying the need for competence. Finally, the achievement of many plans (e.g., social activities, travel) necessarily involves interaction with others, thereby satisfying the need for relatedness. Recent research confirms that satisfying these basic psychological needs is a key pathway for older adults to maintain high levels of subjective well-being (Ryan & Deci, 2024; Tang et al., 2020, 2021). Therefore, planning achievement promotes mental health through a systematic process of psychological need satisfaction. This leads to the following hypothesis:

**H1.**  *Retirement planning achievement positively predicts the mental health of retired older adults.*

### 1.3. Internal Mechanisms of How Retirement Planning Achievement Affects Mental Health: Mediation Pathways

Through what psychological mechanisms does retirement planning achievement enhance mental health? We posit that its influence may not be exerted directly but hinges on fulfilling basic psychological needs. Based on SDT, this study proposes two parallel mediation pathways, corresponding respectively to the satisfaction of the need for relatedness and the needs for autonomy/competence.

**The behavioral pathway: Active Social Participation.** Drawing on the Active Aging framework (World Health Organization, 2021), successful aging is contingent on optimizing conditions for health, participation, and security to expand older adults’ functional capabilities and choices. The focus is not merely on maintaining existing social roles or simply increasing the quantity of activities, but on enhancing the accessibility and quality of participation through inclusive environments and health-promoting policies. This enables older adults to continue contributing, maintain their dignity and well-being, and realize self-determination. Retirement planning achievement is a key enabling factor for social participation, rather than just a simple action script. While past theories (e.g., activity theory) emphasized the importance of participation, they often overlooked its prerequisites. Planning achievement creates these very conditions, transforming the desire to participate into a possibility. For example, successful financial planning provides the economic support for social activities (e.g., travel, paid interest classes, gatherings with friends), removing barriers to social engagement (Rozynek & Lanzendorf, 2023; Moal, 2021). Effective health planning ensures the physical capacity and energy required for dynamic involvement (e.g., physical exercise, community volunteering), which forms the physiological foundation for participation (Palombi et al., 2025). Once these resources, secured through planning achievement, are in place, individuals are better equipped and more confident to initiate and sustain social interactions. From an SDT perspective, this form of social participation—driven by autonomously planned and successfully executed goals—is particularly effective at satisfying the fundamental need for relatedness: the feeling of connection and belonging, which is an indispensable cornerstone of mental health (Ryan & Deci, 2000). For retirees, who face the risk of shrinking social networks after leaving their professional roles, social isolation and loneliness are significant risk factors for depression and anxiety (Santini et al., 2020). Therefore, active social participation is a critical pathway to maintaining and expanding social connections, satisfying the need for relatedness, and combating loneliness (Tang et al., 2021; Levasseur et al., 2015). Numerous recent studies provide strong empirical support, showing that higher frequency and quality of social participation significantly reduce depression and loneliness while increasing life satisfaction and subjective well-being among older adults (Tang et al., 2021; Levasseur et al., 2015).

In summary, retirement planning achievement empowers older adults to engage in more active social participation by providing essential material and health resources. This participation, in turn, fosters strong social connections, effectively satisfying their basic psychological need for relatedness and ultimately promoting mental health (Hu et al., 2025). We therefore hypothesize:

**H2.**  *Active social participation mediates the relationship between retirement planning achievement and mental health.*

**The Psychological Pathway: Retirement Adjustment.** Retirement adjustment refers to the psychological process through which individuals integrate themselves into their new life stage (Dai et al., 2017). In this study, retirement adjustment is operationalized as the combined manifestation of positive and negative psychological experiences after retirement, measured by the two core dimensions in the Retirement Adjustment Questionnaire developed by M.-Y. Zhang and Wang (2018): retirement enjoyment, which captures positive emotions such as satisfaction, pleasure, and a sense of meaning, and retirement loss, which reflects negative experiences such as identity loss and diminished sense of purpose typically resulting from role changes or reduced social engagement. We posit that achieving one’s retirement plan can foster overall adjustment by both enhancing enjoyment and decreasing loss. Beyond being a tangible behavioural outcome, planning achievement shapes individuals’ internal appraisal of retirement—one of life’s major transitions. Retirement adjustment is not a static or singular state, but rather a multifaceted psychological process comprising simultaneous positive and negative dimensions (Hansson et al., 2020; Yuan et al., 2025). It is through improvement in this adjustment process that planning achievement ultimately translates into better mental health.

Self-Determination Theory (SDT) provides the theoretical lens, emphasizing that the satisfaction of autonomy (experiencing one’s behavior as self-determined) and competence (feeling effective in meeting challenges) needs is crucial for intrinsic well-being (Ryan & Deci, 2000; Kiani & Ehsan, 2024). The achievement of retirement plans offers an ideal context for fulfilling these two needs. A retirement plan is a “blueprint” that an individual designs for their own future. When this self-authored blueprint is realized, individuals experience their life trajectory as aligned with their own will and values. This powerfully affirms their personal autonomy, effectively counteracting the potential feelings of lost control or aimlessness that can arise from leaving a mandatory work schedule (Ryan & Deci, 2000; Henning et al., 2025), satisfying the need for autonomy. Successfully implementing a long-term, complex plan is in itself a significant demonstration of efficacy. It proves to the individual their capability to navigate this new and uncertain phase of life, thereby greatly enhancing their self-efficacy and confidence in facing retirement challenges (Wu & Chao, 2024), satisfying the need for competence.

When the needs for autonomy and competence are met through planning achievement, individuals naturally experience higher retirement enjoyment and lower retirement loss. Empirical studies support this, showing that individuals who actively prepare for retirement report a higher quality of life and a smoother adjustment process (Barbosa et al., 2016; Hansson et al., 2020). Conversely, maladjustment, particularly persistent retirement dissatisfaction, has been confirmed by longitudinal research as a significant predictor of future depressive symptoms (Hansson et al., 2020).

In sum, retirement planning achievement fosters positive retirement adjustment (manifested as high enjoyment and low loss) by satisfying individuals’ needs for autonomy and competence, and this well-adjusted state is a core component of overall mental health (Han, 2024). We therefore hypothesize:

**H3.**  *Retirement adjustment mediates the relationship between retirement planning achievement and mental health.*

**H3a.**  *Higher retirement planning achievement predicts greater retirement enjoyment, which in turn leads to better mental health.*

**H3b.**  *Higher retirement planning achievement predicts a lower sense of retirement loss, which in turn leads to better mental health.*

### 1.4. The Moderating Role of Psychological Resources: Retirement Adjustment as an Amplifier of Resource Gains

An individual’s quality of life in retirement depends not only on the achievement of their plans but also on their existing stock of psychological resources. Based on the Conservation of Resources (COR) theory (Hobfoll, 1989; Hobfoll et al., 2018), this study conceptualizes retirement adjustment status as a critical internal psychological resource that can moderate (i.e., amplify or weaken) the positive effects of an external resource injection—retirement planning achievement—on mental health. The core tenet of COR theory is that individuals are inherently motivated to acquire, maintain, and protect their valued resources (Hobfoll et al., 2018). A key corollary of the theory is the “resource gain spiral,” which posits that resource acquisition is not an isolated event but tends to trigger the accumulation of further resources (Jiang et al., 2023). Individuals who are initially “resource-rich” not only have greater reserves to buffer against future losses but, more importantly, are better positioned to leverage newly acquired resources, thereby initiating a positive, self-reinforcing cycle (Hansson et al., 2019; Yeung, 2018). This phenomenon has been widely corroborated in organizational behavior and health psychology: employees with high psychological capital (e.g., optimism, resilience) benefit more from positive work events (Cassanet et al., 2023), and individuals with strong social support can more effectively utilize medical resources when facing health challenges (Barbosa et al., 2016). The value of a resource, therefore, lies not just in its intrinsic worth but in how it interacts with other resources. Consequently, the injection of new resources can have starkly different effects on the “resource-poor” versus the “resource-rich.” For the former, a new resource may merely be used to offset existing deficits, failing to generate significant gains. For the latter, it acts as a catalyst, activating and amplifying the value of their existing resource stock to create a synergistic “1 + 1 > 2” effect (Zhan et al., 2023).

Within the present research framework, retirement planning achievement is considered a significant “resource gain” event, providing retirees with structural (a structured daily routine), conditional (financial security), and personal (a sense of control and accomplishment) resources. However, the efficiency with which these newly acquired resources are converted into enhanced mental health is largely contingent upon the individual’s existing psychological resource stock—their retirement adjustment status.

Retirement enjoyment is the core of positive retirement adjustment, representing the satisfaction, pleasure, and sense of meaning an individual experiences in retirement. Retirees with high enjoyment typically possess more positive emotional states, stronger self-efficacy, and richer social connections (Earl et al., 2015)—all of which are valuable internal psychological resources. According to the COR principle of gain spirals, these “resource-rich” individuals are better equipped to efficiently convert the outcomes of their planning achievement (e.g., more leisure time, financial freedom) into psychological well-being (Hurtado & Topa, 2019). For instance, they might leverage this security to explore new interests and build new social relationships, further consolidating and enhancing their well-being. This positive cycle validates the synergistic and amplifying effect among resources. Thus, we expect retirement enjoyment to positively moderate the relationship between planning achievement and mental health, such that the positive effect is stronger for individuals with higher levels of retirement enjoyment (H4a).

Conversely, Retirement loss reflects negative adjustment and is often associated with a loss of identity, a diminished sense of purpose, and a shrinking social circle (M. Wang et al., 2011; Roosevelt, 2023). Individuals with high retirement loss are in a state of “resource deficit” or a “resource loss spiral.” In this state, their cognitive and emotional resources are depleted in coping with the negative emotions and stress stemming from this loss (Hobfoll et al., 2018). Consequently, even if they achieve their retirement plans, the acquired external resources are likely to be used for “loss offsetting”—filling the psychological void—rather than for “resource investment” in psychological growth. This discounts the positive effects of achievement and may even trigger a negative spiral (Zhan et al., 2023). In other words, only when retirement loss is low (i.e., psychological resource depletion is minimal) do individuals have the “psychological capacity” to fully capitalize on the benefits of their achievements. Thus, we expect retirement loss to negatively moderate the relationship between retirement planning achievement and mental health, such that the positive effect is stronger for individuals with lower levels of retirement loss (H4b).

In summary, the two dimensions of retirement adjustment—enjoyment and loss—collectively represent an individual’s core psychological resource status during the retirement transition. They are not merely outcomes of planning achievement but also act as critical preconditions that regulate the efficiency of converting a new resource (planning achievement) into a final outcome (mental health) (Hansson et al., 2019). This leads to our overall moderation hypothesis:

**H4.**  *Retirement adjustment moderates the relationship between retirement planning achievement and mental health. Specifically:*

**H4a.**  *Retirement enjoyment positively moderates this relationship.*

**H4b.**  *Retirement loss negatively moderates this relationship (i.e., the lower the loss, the stronger the positive impact.*

### 1.5. Socioeconomic Status Moderation: Differences Across Income Levels

All psychological mechanisms are embedded within specific social structures. Household income is the most direct indicator of socioeconomic status, shaping not only the resources individuals can access but also the constraints they face (Shang & Wei, 2023). Insufficient-income groups may prioritize planning for basic survival needs, with their mental health more directly constrained by material conditions (Bakkeli, 2020; Lai et al., 2023). Sufficient-income groups, in contrast, enjoy substantial resource buffers, making the achievement of a single plan less impactful to their overall well-being (Q. Wang & Timonen, 2021). Average-income groups, however, possess both the ability to plan beyond basic needs and yet lack enough reserves to fully buffer risk. Consequently, they are most sensitive to whether planning goals are achieved, and the impact of planning achievement on their psychological state is expected to be most pronounced (C. Liu et al., 2022; X. Liu et al., 2024). This introduces another important question for the present study: Is the pathway from retirement planning achievement to psychological health differentiated by “income poverty” and “income affluence”? Accordingly, we further hypothesize that:

**H5.**  *Overall theoretical model linking retirement planning achievement to mental health differs across household income groups, with the primary effects expected to be most pronounced among the average-income group.*

In addition, based on the theory of subjective socioeconomic status (Subjective SES), individuals’ subjective economic perception often reflects their real-life experiences and social comparison outcomes more accurately than objective income (Adler et al., 2000; Singh-Manoux et al., 2005). Self-rated income has been found to correlate even more strongly with health and well-being than objective measures (Zell et al., 2018), as it captures both actual disposable resources and the affective outcomes of social comparison, rather than nominal earnings alone (Operario et al., 2004; Cheung & Lucas, 2016). Therefore, this study adopts self-rated household income as the predictor variable.

### 1.6. Model Construction and Research Hypotheses

A single variable may simultaneously function as both a mediator and a moderator (Judd et al., 2001). Preacher et al. (2007) developed and validated a similar moderated mediation model (Model 1). Such models were previously described by Judd and Kenny (1981) and framed by James and Brett (1984) as examples of mediation models with moderation. The conceptual structure can also be understood as the path from independent variable X to dependent variable Y being moderated by variable Judd et al. (2001), as well as MacKinnon (2001), discussed the importance of considering such models in scale development.

**Figure 1:**
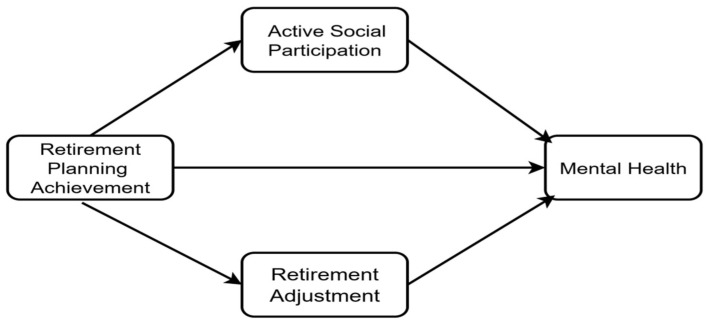
Hypothetical Path Diagram of a Parallel Mediation Model.

**Figure 2:**
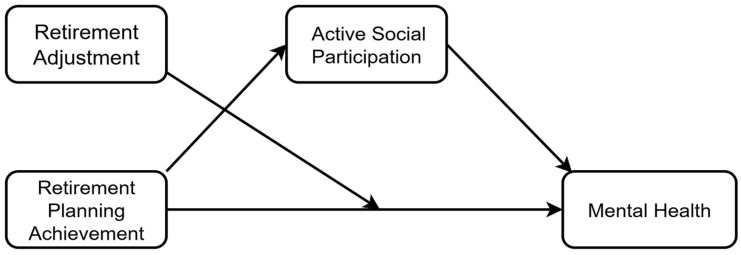
Hypothetical Path Diagram of a Moderated Mediation Model.

Based on this literature, the present study constructs both a parallel mediation model (Figure 1) and a moderated mediation model (Figure 2). First, we test whether retirement planning achievement influences mental health through three parallel mediational pathways: active social participation, retirement enjoyment, and retirement loss (Figure 1). Second, we incorporate retirement adjustment status (retirement enjoyment and retirement loss) as moderating variables (Figure 2) to examine the boundary conditions for the direct effect of planning achievement on mental health. Third, the entire model is tested within groups stratified by household income level, exploring its socioeconomic boundaries.

In summary, this study systematically develops a parallel mediation model and a moderated mediation model to unpack the “black box” linking retirement planning achievement to mental health among retired older adults, and to delineate its socioeconomic boundaries by examining heterogeneity across income levels. The findings will provide empirical evidence for promoting social equity and for the design of targeted, precision aging-support policies.

## 2. Materials and Methods

### 2.1. Participants

An anonymous survey was conducted online via Wenjuan.com (an online survey platform), initially targeting 926 retirees in China aged 55–74. Convenience sampling was used to recruit participants from the platform’s user database, aiming to ensure a degree of representativeness across different genders and age groups. All participants had completed formal retirement procedures and were from various provincial capital cities across the country. After screening responses with lie-detector questions, a final valid sample of 900 was obtained. Demographic characteristics of the sample were as follows: gender evenly distributed (50.0% each); majority aged 55–65 years (72.3%), with 3.9% aged over 70; predominantly married (92.8%); education level concentrated at secondary vocational school or below (70.4%), while 29.6% had a college diploma or above; self-rated household income was rated as average by 64.1% of participants, sufficient by 29.2%, and insufficient by 6.7%; pre-retirement employment was mainly in state-owned enterprises (49.8%) and government/public institutions (21.4%); most held positions as general employees (61.0%) or frontline managers (26.4%); and self-rated health status was distributed as follows: very unhealthy (1.44%), relatively unhealthy (13.44%), average (40.7%), relatively healthy (39.2%), and very healthy (5.22%).

### 2.2. Measures

**Retirement Planning Achievement.** This was measured using the 17-item Retirement Planning Achievement Scale (RPAS) (Wu & Chao, 2024), which comprises five domains: financial, health, social, housing, and psychological. This scale was selected because it was developed and validated within the Chinese cultural context, making it suitable for older adults and demonstrating good reliability and cultural appropriateness. Responses are rated on a 4-point Likert scale (0 = never planned, 1 = planned but not fulfilled, 2 = planned but partially fulfilled, 3 = planned and typically fulfilled), with higher scores indicating a greater degree of achievement. In the present study, the scale showed good internal consistency (Cronbach’s *α* = 0.75).

**Active Social Participation.** This was measured using the 8-item Active Social Participation dimension from the Chinese version of the Active Ageing Scale (J. Zhang et al., 2017). This dimension was chosen as it has been validated in the Chinese cultural context and is well-suited for the Chinese older adult population, showing good reliability and validity. Items are rated on a 4-point Likert scale ranging from 1 (does not apply at all) to 4 (applies completely), with higher scores indicating greater levels of social participation. The Cronbach’s *α* for this dimension in the present study was 0.84.

**Retirement Adjustment.** This was assessed using two dimensions—retirement enjoyment and retirement loss—from the Chinese version of the Retirement Adjustment Questionnaire (M.-Y. Zhang & Wang, 2018). This questionnaire was selected because it is a well-established and authoritative instrument for measuring retirement adjustment in China. Its enjoyment and loss dimensions effectively capture the core positive and negative experiences of retirement, aligning well with this study’s theoretical constructs. All items were rated on a 5-point Likert scale (1 = strongly disagree, 5 = strongly agree). Scores on the retirement loss dimension were reverse-coded, so that higher scores reflected lower feelings of loss and better adjustment. In the present study, the Cronbach’s *α* coefficients for retirement enjoyment and retirement loss were 0.71 and 0.68, respectively.

**Mental Health.** This was assessed using the 20-item Short Form of the Mental Health Scale for Older Adults (Fu et al., 2023). The scale was chosen because it was specifically developed and validated for the Chinese older adult population within their cultural context, ensuring its suitability and psychometric soundness. It comprises five dimensions: adaptability, cognitive efficacy, interpersonal relationships, emotional experience, and self-perception. Items are scored on a 4-point Likert scale (1 = does not apply, 4 = applies), with higher scores indicating better mental health. The scale’s Cronbach’s *α* in this study was 0.74.

### 2.3. Data Analysis

**Figure 3:**
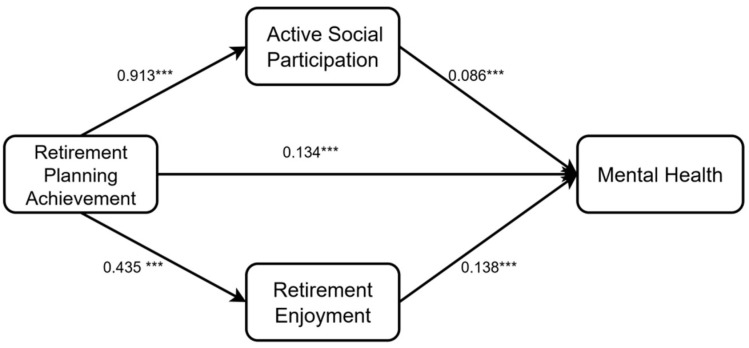

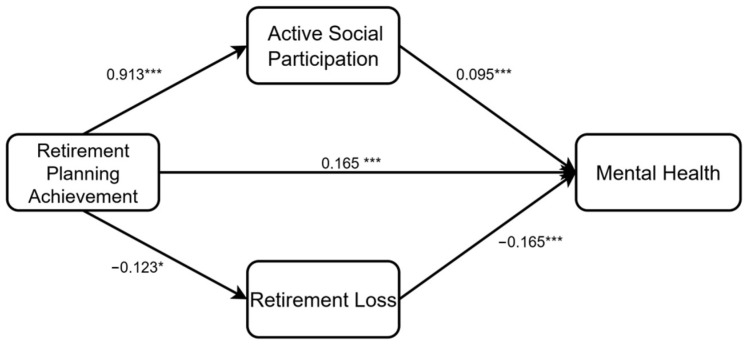
Path Analysis Results of the Parallel Mediation Model. Note: * *p* < 0.05; *** *p* < 0.001.

This study employed a two-step analysis strategy to test the hypothesized model, with data analysis conducted using SPSS 29.0 and Mplus 8.3. First, a path analysis using structural equation modeling (SEM) was performed in Mplus 8.3 to examine the overall goodness-of-fit of the model (see Section 3.2 and Figure 3). This step aimed to confirm at a macro level that the proposed theoretical model had a good fit with the data. Second, to more precisely test the specific mediation and moderated mediation effects, the SPSS PROCESS macro was used (Model 4 and Model 5), following Hayes (2017). Bootstrap resampling was set to 5000 to obtain bias-corrected 95% confidence intervals, as the PROCESS macro provides more robust tests for the significance of specific indirect and conditional indirect effects. The results from these two complementary methods provide rigorous statistical support for the study’s hypotheses. All main analyses controlled for demographic variables, including gender, age, marital status, and educational level. Finally, it should be noted that this study employed a cross-sectional design to preliminarily explore the complex relationships among variables; therefore, causal inferences should be drawn with caution.

## 3. Results

### 3.1. Common Method Bias Test and Correlation Analysis

Harman’s single-factor test was used to examine potential common method bias. Unrotated exploratory factor analysis extracted multiple factors with eigenvalues greater than 1, with the first factor accounting for 15.33% of the total variance—well below the 40% threshold (Podsakoff et al., 2003). This suggests that serious common method bias was not present in the data.

Descriptive statistics and correlations for key variables (Table 1 and Table 2) showed that the directions of the associations were consistent with theoretical expectations, providing a sound basis for subsequent hypothesis testing.

### 3.2. Test of Parallel Mediation Effects

A parallel mediation model was constructed using Mplus 8.3, with retirement planning achievement as the independent variable (x), mental health as the dependent variable (y), active social participation (m1), retirement enjoyment (w1), and reverse-scored retirement loss (w2) as mediators. All demographic variables were included as covariates. Model fit indices indicated good fit (Table 3). The path analysis results are shown in Figure 3.

Bootstrapping procedures with 5000 resamples using the PROCESS macro (Model 4) (Table 4 and Table 5) showed that the total indirect effect of retirement planning achievement on mental health was 0.28 (95% *CI*: 0.23 to 0.33), excluding zero. When all mediators were included, the direct effect ranged from 0.12 to 0.16, accounting for 44.40–55.96% of the total effect, with the 95% *CI* excluding 0. **These findings supported H1**.

Retirement planning achievement indirectly influenced mental health through three pathways:

- **Active social participation:** indirect effect = 0.09–0.01, accounting for 32.85–35.38% of the total effect (95% *CI* excluded 0).
- **Retirement enjoyment (H3a):** indirect effect = 0.06, accounting for 22.38% of the total effect (95% *CI:* 0.04 to 0.09).
- **Retirement loss (H3b):** indirect effect = 0.02, accounting for 8.30% of the total effect (95% *CI*: 0.00 to 0.05), but in the opposite sign. Specifically, retirement planning achievement negatively predicted retirement loss (higher scores = lower loss), which in turn negatively predicted mental health. Multiplying the two negative paths yielded a positive indirect effect.

**These results supported H2 and H3.**

Although both Mplus 8.3 and PROCESS used 5000 bootstrap resamples, slight differences in the estimated effects were observed. These arise mainly from methodological differences: SEM in Mplus models latent variables with maximum likelihood while accounting for measurement error, whereas PROCESS applies OLS regression to observed variables. Minor variations in model specification and standardization may explain the small discrepancies, but the consistent direction and significance of the effects indicate the robustness of the findings.

### 3.3. Test of Moderation Effects

A moderated mediation model was tested using the PROCESS macro (Model 5), with retirement planning achievement as x, mental health as y, active social participation (m1) as the mediator, and retirement enjoyment (w1) and reverse-scored retirement loss (w2) as moderators (Figure 2). All demographic variables were entered as covariates.

#### 3.3.1. Moderating Effect of Retirement Enjoyment

**Figure 4:**
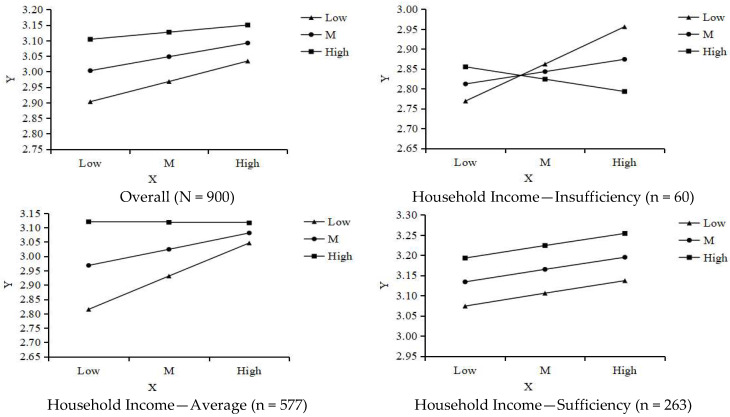
Moderating Effect of Retirement Enjoyment. Note: High = M + 1SD; Low = M − 1SD.

As shown in Table 6, the interaction between retirement planning achievement and retirement enjoyment (Int_1) significantly and negatively predicted mental health (*B* = −0.096, *p* < 0.01, 95% *CI*: −0.166 to −0.026). Simple slope analysis (Figure 4, Table 7) indicated that for individuals with low retirement enjoyment (*M* − 1*SD*), the positive effect of retirement planning achievement on mental health was strongest (Effect = 0.17, *p* < 0.001). In contrast, for individuals with high retirement enjoyment (*M* + 1*SD*), the effect was weaker and nonsignificant (Effect = 0.06, *p* = 0.13). This pattern contradicts the “amplification effect” predicted in H4a and instead suggests a “ceiling effect” or “need compensation” effect, whereby the benefits of retirement planning achievement are greater for those who derive less enjoyment from retirement life. Thus, H4a was not supported, although a significant negative moderation effect was identified.

#### 3.3.2. Moderating Effect of Retirement Loss

**Figure 5:**
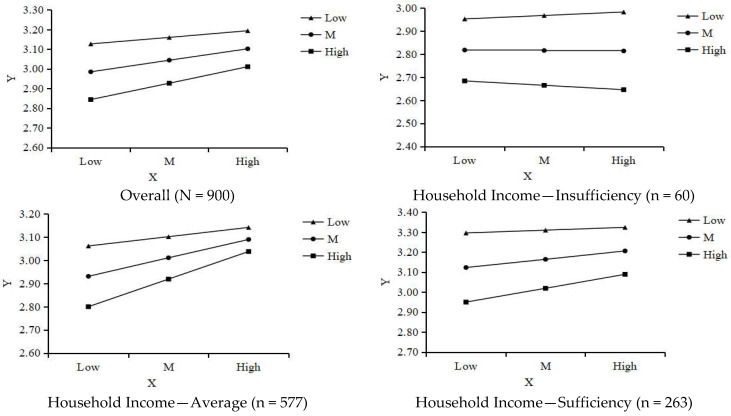
Moderating Effect of Retirement Loss. Note: High = M + 1SD; Low = M − 1SD.

Table 8 indicates that the interaction between retirement planning achievement and reverse-scored retirement loss (Int_2) significantly and positively predicted mental health (*B* = 0.094, *p* < 0.01, 95% *CI*: 0.034 to 0.154). Because retirement loss was reverse-scored (higher scores = lower loss), this positive interaction implies that the beneficial effect of retirement planning achievement on mental health becomes stronger as retirement loss decreases. Simple slope analysis (Figure 5, Table 9) confirmed this: in the low-loss (high-score, *M* + 1*SD*) group, the effect was strongest (Effect = 0.22, *p* < 0.001); in the high-loss (low-score, *M* − 1*SD*) group, the effect was significantly weaker (Effect = 0.09, *p* < 0.02). These findings align fully with H4b, supporting the hypothesized “amplification effect.”

### 3.4. Differential Effects by Household Income Level

Subgroup analyses were conducted to examine mediation and moderation effects across household income levels, thus revealing socio-economic boundaries of the proposed model.

- **Socioeconomic Differences in Mediation Effects** (Table 4 and Table 5): For the insufficient-income group, both the direct effect and most indirect pathways were found to be non-significant. In the average-income group, all direct and indirect effects were significant, yielding high explanatory power for the model. For the sufficient-income group, the total effect and most indirect effects remained significant; however, the direct effect was non-significant in the model that included retirement enjoyment (m1_w1) (*p* = 0.076), as was the mediating pathway through reduced retirement loss (95% *CI* included 0).
- **Socioeconomic Differences in Moderation Effects** (Table 6, Table 7, Table 8 and Table 9): Both significant moderation effects, negative moderation by retirement enjoyment and positive moderation by reverse-scored retirement loss, were observed almost entirely in the average-income group (all interaction terms *p* < 0.001). In the insufficient-income and sufficient-income groups, interaction terms were nonsignificant. Although the overall interaction effect of retirement loss was nonsignificant in the sufficient-income group, further analysis (Figure 5, Table 9) indicated that for members of this group with medium-to-high retirement loss scores, retirement planning achievement still exerted a significant positive effect on mental health. This suggests that a moderation pattern may persist, albeit in attenuated form.

In summary, these findings provide strong support for H5. The complex psychological mechanism linking retirement planning achievement, active social participation/adjustment, and mental health shows clear socio-economic stratification, functioning most robustly among retired older adults with average household income, while being substantially weakened or no longer applicable at both the lower and higher ends of the socio-economic spectrum. In the insufficient-income group, nearly all mediation and moderation effects were non-significant. However, given the relatively small sample size of this subgroup (n = 60), the statistical power may have been limited; thus, these non-significant results should be interpreted with caution.

## 4. Discussion

This study employed a progressive model-testing strategy to systematically reveal the mechanisms by which retirement planning achievement influences the mental health of older adults, as well as the socioeconomic boundaries of these effects. The results not only confirmed the direct and positive impact of retirement planning achievement on mental health, but, more importantly, by constructing both parallel mediation and moderated mediation models, also elucidated the internal pathways and situational contingencies underlying this relationship. These findings make important theoretical contributions to our understanding of positive aging.

### 4.1. Theoretical Contributions and Model Innovations

First, this study shifts the academic lens from the traditional emphasis on “retirement planning behavior” to the level of “retirement planning achievement,” highlighting that it is the achievement—rather than merely the intention or the act itself—that serves as the true predictor of mental health. This conceptual shift draws upon goal attainment theory and psychological contract theory, viewing planning achievement as the fulfillment of a contract between the individual and their future self (Rousseau, 1995; Locke & Latham, 1990). Our findings validate the view that the subjective sense of efficacy, autonomy, and control resulting from the achievement of retirement plans far exceeds the psychological benefits of planning alone (Bandura, 1997; Wu & Chao, 2024). This underscores that it is the implementation, rather than the plan itself, that empowers older individuals to experience subjective well-being. Such insights suggest an important direction for refining aging-related policies, moving beyond a focus on planning intentions or single behavioral components (J. H. Noone et al., 2009; Yeung & Zhou, 2017).

### 4.2. Systematic Elucidation of Psychological Mechanisms

Drawing on Self-Determination Theory (SDT) as the core analytical framework (Deci & Ryan, 2000), this study is among the first to empirically differentiate and test two parallel mediational pathways: the fulfillment of relatedness needs (via active social participation) and the satisfaction of autonomy and competence needs (via retirement adjustment—namely, increased enjoyment and reduced loss). The results indicate that retirement planning achievement facilitates active social participation, effectively satisfying the need for relatedness, enriching social networks, and reducing loneliness and depression (Tang et al., 2021; Levasseur et al., 2015). At the same time, it enhances autonomy and competence by increasing retirement enjoyment and reducing retirement loss, thus strengthening self-efficacy and positive self-evaluation (Ryan & Deci, 2024). This multi-path mechanism validates SDT and its subsequent extensions in the realm of older adults’ mental health, providing fresh empirical support for a cohesive theoretical integration (Tang et al., 2020).

### 4.3. The Moderating Role of Psychological Resources: Resource Gain Beyond Compensation

Departing from the classic stress-buffering perspective (which treats vulnerable groups as the main beneficiaries; Hobfoll, 1989), this study reveals that the resource gain effect of retirement adjustment status—especially low retirement loss—is particularly pronounced. That is, well-adapted individuals can more efficiently translate planning achievement into mental health gains, producing a “more than the sum of its parts” resource spiral effect (Hansson et al., 2019; Jiang et al., 2023). Conversely, among those with depleted psychological resources (high retirement loss), even retirement planning achievement yields only limited benefits. This insight suggests a need for more targeted support services: interventions that combine external support with a base of strong subjective resources may yield multiplicative psychological returns (Zhan et al., 2023). Interestingly, retirement enjoyment exhibited a ceiling effect as a moderator: when retirees’ enjoyment is already high, the marginal effects of further planning achievement on mental health are diminished—indicating limited scope for external intervention to further boost well-being in this group (Han & Wang, 2022). This ceiling pattern contrasts theoretically with the inhibiting effect of high retirement loss, reflecting the differentiated roles that facets of psychological need satisfaction play in shaping late-life well-being.

### 4.4. Socioeconomic Stratification and Heterogeneity of Psychological Mechanisms

This study further highlights striking heterogeneity in the model effects across income strata: the primary effects were most salient and robust in the average-income group, be it in the mediation or moderation processes. For insufficient-income individuals, unmet basic survival and security needs constrain the scope for psychological resources or planning achievement to interact meaningfully (Maslow, 1943; Bakkeli, 2020). For sufficient-income retirees, abundant resources buffer the potential impact of any single achieved plan (Q. Wang & Timonen, 2021). In contrast, for average-income individuals, the intersection of retirement planning achievement and psychological resources translates most efficiently into subjective well-being (X. Liu et al., 2024; Lai et al., 2023), suggesting both the unique policy relevance and latent psychological vulnerability of the emerging middle class in the context of healthy aging. These findings align with health inequality and subjective socioeconomic status theories, which emphasize the stratification of psychological well-being (Adler et al., 2000; Hoogendijk et al., 2018), and provide a crucial empirical foundation for understanding the dynamics of social mobility in China’s ongoing economic transformation.

### 4.5. Dual Roles of Retirement Adjustment: Mediation and Moderation

This study conceptualizes retirement adjustment as both a mediator and a moderator, reflecting its multifaceted function in cross-sectional data. From a longitudinal perspective, these relations may unfold as a dynamic process: early planning achievement fosters initial positive adjustment (mediation), and this emergent positive adjustment—operating as a psychological resource—subsequently amplifies the beneficial impact of later life events (including continued planning achievement) on mental health (moderation). Future longitudinal research should further examine this dynamic, recursive process.

### 4.6. Practical Implications and Policy Recommendations

This study provides crucial practical insights for improving retiree mental health. The central takeaway is that policies and services must strategically shift focus from pre-retirement planning to post-retirement achievement. Since realizing plans is key to well-being, support must continue after retirement. Continuous assistance, like plan-tracking apps, peer-sharing forums, or community advisors, is essential to help retirees translate plans into reality. Moreover, these interventions should be targeted for maximum impact, particularly on the average-income group, who are most psychologically responsive to planning achievement, making such efforts highly cost-effective. Empowering this group through self-efficacy and peer support can activate their high-sensitivity advantage to better tackle the societal mindset challenges of an aging population (Shang & Wei, 2023). Lastly, interventions should follow a mitigate loss before promoting gain principle. Our findings show that a low sense of loss powerfully amplifies positive outcomes. Therefore, the first step should be to help retirees mitigate feelings of loss via counseling, support groups, or new hobbies. Once this foundational psychological deficit is addressed, subsequent gain-oriented interventions that encourage planning achievement will be far more impactful and yield the best results.

### 4.7. Limitations and Future Directions

This study is not without limitations. Its cross-sectional design limits causal inference; the reliability of the retirement loss subscale (α = 0.681) is only marginal (Nunnally, 1978); the self-reported household income may be subject to social desirability bias; and the study does not distinguish between different types of planning achievements. Furthermore, the study faces issues of sample representativeness, particularly the small sample size of the insufficient-income group (n = 60), which limited the statistical power for testing complex models, thus, we cannot definitively conclude that the proposed psychological mechanism is absent in this group, only that our study lacked the power to detect it. Therefore, future research should employ longitudinal designs, refine measurement tools, incorporate objective economic data, and—crucially—utilize more refined stratified sampling or targeted recruitment to obtain larger, more balanced samples. This would allow for a more robust validation or revision of our preliminary findings concerning the moderating role of income level.

## 5. Conclusions

Retirement planning achievement is an important pathway for enhancing the mental health of older adults. Its mechanisms operate through promoting active social participation (which fulfills relatedness needs) and improving retirement adjustment (which satisfies needs for autonomy and competence). However, these “planning dividends” are not universal: their positive effects are significantly more pronounced among retirees with better adjustment (amplifier effect), and this psychological mechanism applies primarily to average-income retired groups. These findings profoundly reveal the complexity and heterogeneity of the retirement adjustment process and its socioeconomic logic and carry important theoretical and practical implications for building more targeted and equitable systems of aging support.

## Figures and Tables

**Table 1:** Partial Correlations Among Core Variables and Their Subdimensions.

Variable	1	2	3	4	5	6
1 Retirement Planning Achievement	1					
2 Active Social Participation	0.57 ***	1				
3 Retirement Enjoyment	0.29 ***	0.10 **	1			
4 Retirement Loss	−0.07 *	0.02	−0.34 ***	1		
5 Nostalgia for Work	0.11 **	0.14 ***	−0.20 ***	0.44 ***	1	
6 Mental Health	0.34 ***	0.31 ***	0.33 ***	−0.39 ***	−0.10 **	1

Note: * *p* < 0.05; ** *p* < 0.01; *** *p* < 0.001.

**Table 2:** Means, Standard Deviations, and Correlation Coefficients of the Variables.

	*M*	*SD*	1	2	3	4	5	6	7	8	9	10	11	12	13	14	15
1 Gender	1.50	0.50	1														
2 Age groups	2.84	1.35	−0.32 ***	1													
3 Marital Status	1.17	0.63	0.07 *	0.01	1												
4 Educational attainment	2.00	1.00	0.04	−0.21 ***	0.01	1											
5 Household Income	2.23	0.56	0.04	0.01	0.03	0.22 ***	1										
6 Type of pre-retirement employer	2.07	0.71	0.03	−0.04	0.02	−0.16 ***	−0.01	1									
7 Pre-retirement position	1.52	0.71	−0.02	0.02	0.00	0.61 ***	0.34 ***	−0.08 *	1								
8 Years since retirement	2.10	0.86	0.18 ***	0.63 ***	0.03	−0.20 ***	−0.01	0.01	−0.03	1							
9 Self-rated Health	3.33	0.83	0.06	−0.09 **	0.03	0.11 ***	0.26 ***	0.04	0.13***	−0.07 *	1						
10 Retirement Planning Achievement	2.01	0.39	0.10 **	−0.04	−0.04	0.15 ***	0.38 ***	−0.01	0.19 ***	−0.05	0.18 ***	1					
11 Active Social Participation	2.72	0.63	0.13 ***	−0.09 **	0.02	0.14 ***	0.36 ***	0.11 **	0.19 ***	−0.02	0.18 ***	0.63 ***	1				
12 Retirement Enjoyment	4.08	0.57	0.10 **	−0.14 ***	−0.04	0.10 **	0.23 ***	−0.09 **	0.10**	−0.05	0.20 ***	0.36 ***	0.19 ***	1			
13 Retirement Loss	2.93	0.70	0.02	0.07 *	−0.04	−0.09 **	−0.24 ***	0.07 *	−0.10**	0.07 *	−0.13 ***	−0.16 ***	−0.06	−0.39 ***	1		
14 Nostalgia for Work	3.28	1.01	−0.01	0.06	−0.03	0.02	−0.02	−0.03	0.05	0.03	−0.00	0.10 **	0.12 ***	−0.19 ***	0.43 ***	1	
15 Mental Health	3.04	0.31	0.01	−0.03	−0.11 **	0.06	0.30 ***	−0.06	0.12***	−0.07 *	0.22 ***	0.42 ***	0.37 ***	0.39 ***	−0.43 ***	−0.09 **	1

Note: * p < 0.05; ** p < 0.01; *** p < 0.01.

**Table 3:** Results of Model Fit Analysis.

Measurement Model	*ML χ* ^2^	*df*	*χ*^2^/*df*	*CFI*	*TLI*	*RMSEA*	*SRMR*
Hypothetical Model (m1_w1)	31.068	9	3.452	0.978	0.918	0.073	0.022
Hypothetical Model (m1_w2)	29.346	9	3.261	0.979	0.924	0.071	0.024

Note: CFI = Comparative Fit Index; TLI = Tucker–Lewis index; SRMR = Standardized Root Mean Square Residual; RMSEA = Root Mean Square of Approximation.

**Table 4:** Tests of the Mediation Effects of Active Social Participation and Retirement Enjoyment.

			Effect Value	Boot SE	*t*	*p*	Boot LLCI	Boot ULCI	Effectiveness Ratio (%)
Overall (N = 900)		Total effect	0.28	0.03	10.61	0.00	0.23	0.33	
		Direct effects	0.12	0.03	3.92	0.00	0.06	0.18	44.40
		Indirect effects	0.15	0.02			0.11	0.20	55.60
		Indirect effects m1	0.09	0.02			0.06	0.13	32.85
		Indirect effects w1	0.06	0.01			0.04	0.09	22.38
		m1 − w1	0.029	0.02			−0.01	0.07	
Household Income	Insufficiency (n = 60)	Total effect	0.25	0.09	2.65	0.01	0.06	0.44	
		Direct effects	0.09	0.15	0.57	0.57	−0.21	0.38	34.14
		Indirect effects	0.16	0.15			−0.11	0.47	65.86
		Indirect effects m1	0.17	0.13			−0.07	0.46	67.87
		Indirect effects w1	−0.01	0.04			−0.09	0.09	−2.01
		m1 − w1	0.17	0.14			−0.06	0.46	
	Average (n = 577)	Total effect	0.31	0.03	9.48	0.00	0.25	0.37	
		Direct effects	0.16	0.04	4.01	0.00	0.08	0.24	51.46
		Indirect effects	0.15	0.03			0.10	0.21	48.87
		Indirect effects m1	0.08	0.02			0.04	0.13	25.57
		Indirect effects w1	0.07	0.02			0.04	0.11	22.98
		m1 − w1	0.01	0.03			−0.05	0.06	
	Sufficiency (n = 263)	Total effect	0.27	0.06	4.70	0.00	0.16	0.39	
		Direct effects	0.11	0.06	1.78	0.08	−0.01	0.24	41.33
		Indirect effects	0.16	0.04			0.09	0.25	58.67
		Indirect effects m1	0.11	0.04			0.05	0.18	39.48
		Indirect effects w1	0.05	0.02			0.02	0.10	19.19
		m1 − w1	0.06	0.04			−0.03	0.14	

**Table 5:** Tests of the Mediation Effects of Active Social Participation and Retirement Loss.

			Effect Value	Boot SE	*t*	*p*	Boot LLCI	Boot ULCI	Effectiveness Ratio (%)
Overall (N = 900)		Total effect	0.28	0.03	10.61	0.00	0.23	0.33	
		Direct effects	0.16	0.03	5.40	0.00	0.10	0.21	55.96
		Indirect effects	0.12	0.02			0.08	0.16	43.68
		Indirect effects m1	0.10	0.02			0.07	0.13	35.38
		Indirect effects w2	0.02	0.01			0.00	0.05	8.30
		m1 − w2	0.08	0.02			0.03	0.11	
Household Income	Insufficiency (n = 60)	Total effect	0.25	0.09	2.65	0.01	0.06	0.44	
		Direct effects	−0.02	0.13	−0.12	0.91	−0.27	0.24	−6.02
		Indirect effects	0.26	0.12			0.04	0.50	106.02
		Indirect effects m1	0.20	0.11			0.00	0.43	79.52
		Indirect effects w2	0.07	0.05			−0.02	0.18	26.51
		m1 − w2	0.13	0.12			−0.09	0.39	
	Average (n = 577)	Total effect	0.31	0.03	9.48	0.00	0.25	0.37	
		Direct effects	0.21	0.04	5.43	0.00	0.13	0.28	66.99
		Indirect effects	0.10	0.02			0.06	0.15	33.01
		Indirect effects m1	0.08	0.02			0.04	0.12	25.24
		Indirect effects w2	0.02	0.01			0.00	0.05	7.77
		m1 − w2	0.05	0.02			0.01	0.10	
	Sufficiency (n = 263)	Total effect	0.27	0.06	4.70	0.00	0.16	0.39	
		Direct effects	0.14	0.05	2.71	0.01	0.04	0.24	51.29
		Indirect effects	0.13	0.05			0.05	0.23	48.71
		Indirect effects m1	0.13	0.03			0.07	0.20	47.60
		Indirect effects w2	0.00	0.03			−0.05	0.07	1.48
		m1 − w2	0.13	0.04			0.04	0.22	

**Table 6:** Regression and Subgroup Analyses of the Moderating Effect of Retirement Enjoyment.

Variable	Model (m1)					Model (y)				Household Income (Mode l (y))											
	Overall (N = 900)									Insufficiency (n = 60)				Average (n = 577)				Sufficiency (n = 263)			
	*B*	*SE*	95% *CI*		*B*		*SE*	95% *CI*		*B*	*SE*	95% *CI*		*B*	*SE*	95% *CI*		*B*	*SE*	95% *CI*	
			*LLCI*	*ULCI*				*LLCI*	*ULCI*			*LLCI*	*ULCI*			*LLCI*	*ULCI*			*LLCI*	*ULCI*
x	0.92 ***	0.05	0.84	1.01	0.115 ***		0.03	0.05	0.18	0.07	0.15	−0.23	0.37	0.16 ***	0.04	0.08	0.23	0.11	0.06	−0.01	0.24
m1					0.10 ***		0.02	0.06	0.14	0.21 *	0.10	0.00	0.42	0.08 ***	0.02	0.04	0.12	0.13 ***	0.03	0.07	0.20
w1					0.14 ***		0.02	0.11	0.17	−0.03	0.07	−0.17	0.11	0.17 ***	0.02	0.13	0.21	0.12 ***	0.03	0.06	0.18
Int_1					−0.10 **		0.04	−0.17	−0.03	−0.20	0.13	−0.47	0.07	−0.29 ***	0.05	−0.39	−0.19	0.00	0.11	−0.22	0.22
*R* ^2^	0.434				0.309					0.419				0.302				0.257			
*F*	68.071 ***				30.414 ***					2.823 **				20.37 ***				7.201 ***			

Note: * *p* < 0.05; ** *p* < 0.01; *** *p* < 0.001.

**Table 7:** Moderating Effect of Retirement Enjoyment on the Relationship Between Retirement Planning Achievement and Mental Health.

	*M* ± *SD*	*Effect*	*SE*	*t*	*p*	*LLCI*	*ULCI*
Overall (N = 900)	−0.57	0.17	0.04	4.74	0.00	0.10	0.24
	0.00	0.12	0.03	3.66	0.00	0.05	0.18
	0.57	0.06	0.04	1.54	0.13	−0.02	0.14
Insufficiency (n = 60)	−0.68	0.21	0.17	1.23	0.22	−0.13	0.54
	0.00	0.07	0.15	0.47	0.64	−0.23	0.37
	0.68	−0.07	0.18	−0.38	0.70	−0.43	0.29
Average (n = 577)	−0.56	0.32	0.05	6.63	0.00	0.22	0.41
	0.00	0.16	0.04	4.03	0.00	0.08	0.23
	0.56	−0.01	0.05	−0.12	0.90	−0.10	0.09
Sufficiency (n = 263)	−0.50	0.11	0.09	1.33	0.19	−0.05	0.28
	0.00	0.11	0.06	1.78	0.08	−0.01	0.24
	0.50	0.11	0.08	1.33	0.18	−0.05	0.28

**Table 8:** Regression and Subgroup Analyses of the Moderating Effect of Retirement Loss.

Variable	Model (m1)				Model (y)				Household Income (Model (y))											
	Overall (N = 900)								Insufficiency (n = 60)				Average (n = 577)				Sufficiency (n = 263)			
	*B*	*SE*	95% *CI*		*B*	*SE*	95% *CI*		*B*	*SE*	95% *CI*		*B*	*SE*	95% *CI*		*B*	*SE*	95% *CI*	
			*LLCI*	*ULCI*			*LLCI*	*ULCI*			*LLCI*	*ULCI*			*LLCI*	*ULCI*			*LLCI*	*ULCI*
x	0.92 ***	0.05	0.84	1.01	0.15 ***	0.03	0.10	0.21	0.00	0.13	−0.27	0.26	0.22 ***	0.04	0.14	0.29	0.15 **	0.05	0.05	0.25
m1					0.11 ***	0.02	0.07	0.14	0.18 *	0.09	0.00	0.37	0.08 ***	0.02	0.04	0.12	0.16 ***	0.03	0.10	0.21
w2					−0.17 ***	0.01	−0.19	−0.14	−0.23 **	0.07	−0.36	−0.10	−0.15 ***	0.02	−0.18	−0.11	−0.19 ***	0.02	−0.22	−0.16
Int_2					0.09 **	0.03	0.03	0.15	−0.06	0.14	−0.33	0.22	0.17 ***	0.05	0.08	0.27	0.13	0.07	0.00	0.26
*R* ^2^	0.434				0.378				0.516				0.282				0.487			
*F*	68.071 ***				41.443 ***				4.177 ***				18.491 ***				19.788 ***			

Note: * *p* < 0.05; ** *p* < 0.01; *** *p* < 0.001.

**Table 9:** Moderating Effect of Retirement Loss on the Relationship Between Retirement Planning Achievement and Mental Health.

	*M* ± *SD*	*Effect*	*SE*	*t*	*p*	*LLCI*	*ULCI*
Overall (N = 900)	−0.70	0.09	0.04	2.36	0.02	0.01	0.16
	0.00	0.15	0.03	5.29	0.00	0.10	0.21
	0.70	0.22	0.04	6.21	0.00	0.15	0.29
Insufficiency (n = 60)	−0.67	0.03	0.17	0.19	0.85	−0.32	0.38
	0.00	0.00	0.13	−0.03	0.97	−0.27	0.26
	0.67	−0.04	0.14	−0.29	0.78	−0.33	0.25
Average (n = 577)	−0.63	0.11	0.05	2.38	0.02	0.02	0.20
	0.00	0.22	0.04	5.77	0.00	0.14	0.29
	0.63	0.33	0.05	6.58	0.00	0.23	0.43
Sufficiency (n = 263)	−0.78	0.05	0.07	0.72	0.47	−0.09	0.19
	0.00	0.15	0.05	2.94	0.00	0.05	0.25
	0.78	0.25	0.08	3.24	0.00	0.10	0.41

## Data Availability

Requests for data can be sent to the corresponding author.

## References

[B1-behavsci-15-01593] Adler N. E., Epel E. S., Castellazzo G., Ickovics J. R. (2000). Relationship of subjective and objective social status with psychological and physiological functioning: Preliminary data in healthy, White women. Health Psychology.

[B2-behavsci-15-01593] Bakkeli N. Z. (2020). Older adults’ mental health in China: Examining the relationship between income inequality and subjective wellbeing using panel data analysis. Journal of Happiness Studies.

[B3-behavsci-15-01593] Bandura A. (1997). Self-efficacy: The exercise of control.

[B4-behavsci-15-01593] Barbosa L. M., Monteiro B., Murta S. G. (2016). Retirement adjustment predictors—A systematic review. Work, Aging and Retirement.

[B5-behavsci-15-01593] Cassanet A., McKenzie W. A., McLean L. A. (2023). Psychosocial interventions to support retirement well-being and adjustment: A systematic review. Educational and Developmental Psychologist.

[B6-behavsci-15-01593] Cheung F., Lucas R. E. (2016). Income inequality is associated with stronger social comparison effects: The effect of relative income on life satisfaction. Journal of Personality and Social Psychology.

[B7-behavsci-15-01593] Dai Y. E., Wen F. F., Zuo B., Wu Y., Dai T. T. (2017). Individual-based psychological models of retirement. Advances in Psychological Science.

[B8-behavsci-15-01593] Deci E. L., Ryan R. M. (2000). The “what” and “why” of goal pursuits: Human needs and the self-determination of behavior. Psychological Inquiry.

[B9-behavsci-15-01593] Earl J. K., Gerrans P., Halim V. A. (2015). Active and adjusted: Investigating the contribution of leisure, health and psychosocial factors to retirement adjustment. Leisure Sciences.

[B10-behavsci-15-01593] Emmons R. A. (1986). Personal strivings: An approach to personality and subjective well-being. Journal of Personality and Social Psychology.

[B11-behavsci-15-01593] Feldman D. C., Beehr T. A. (2011). A three-phase model of retirement decision making. American Psychologist.

[B12-behavsci-15-01593] Festinger L. (1957). A theory of cognitive dissonance.

[B13-behavsci-15-01593] Fu J.-N., Zhai B.-Y., Zhao X.-F., Zheng Z.-W., Li J. (2023). Reliability and validity of the brief version of the mental health inventory for the elderly. Chinese Journal of Clinical Psychology.

[B14-behavsci-15-01593] Han B. X. (2024). Psychological adjustment of retirement.

[B15-behavsci-15-01593] Han B. X., Wang X. R. (2022). From flow to flourishing: Comprehensive models of happiness. Journal of Soochow University (Educational Science Edition).

[B16-behavsci-15-01593] Hansson I., Buratti S., Johansson B., Berg A. I. (2019). Beyond health and economy: Resource interactions in retirement adjustment. Aging & Mental Health.

[B17-behavsci-15-01593] Hansson I., Buratti S., Thorvaldsson V., Johansson B., Berg A. I. (2020). Disentangling the mechanisms of retirement adjustment: Determinants and consequences of subjective well-being. Work, Aging and Retirement.

[B18-behavsci-15-01593] Hayes A. F. (2017). Introduction to mediation, moderation, and conditional process analysis: A regression-based approach.

[B19-behavsci-15-01593] Henning G., Segel-Karpas D., Hyde M., Huxhold O. (2025). Retirement adjustment in the pandemic—Did risk-and protective factors change?. Social Indicators Research.

[B20-behavsci-15-01593] Hobfoll S. E. (1989). Conservation of resources: A new attempt at conceptualizing stress. American Psychologist.

[B21-behavsci-15-01593] Hobfoll S. E. (2001). The influence of culture, community, and the nested—Self in the stress process: Advancing conservation of resources theory. Applied Psychology.

[B22-behavsci-15-01593] Hobfoll S. E., Halbesleben J., Neveu J. P., Westman M. (2018). Conservation of resources in the organizational context: The reality of resources and their consequences. Annual Review of Organizational Psychology and Organizational Behavior.

[B23-behavsci-15-01593] Hoogendijk E. O., Rijnhart J. J. M., Kowal P., Pérez-Zepeda M. U., Cesari M., Abizanda P., Ruano T. F., Schop-Etman A., Huisman M., Dent E. (2018). Socioeconomic inequalities in frailty among older adults in six low-and middle-income countries: Results from the WHO Study on global AGEing and adult health (SAGE). Maturitas.

[B24-behavsci-15-01593] Hu Y.-L., Yao J.-Y., Hu Z.-D. (2025). A study of the impact of social participation on the mental health of the elderly in China. Northwest Population Journal.

[B25-behavsci-15-01593] Hurtado M. D., Topa G. (2019). Quality of life and health: Influence of preparation for retirement behaviors through the serial mediation of losses and gains. International Journal of Environmental Research and Public Health.

[B26-behavsci-15-01593] James L. R., Brett J. M. (1984). Mediators, moderators, and tests for mediation. Journal of Applied Psychology.

[B27-behavsci-15-01593] Jiang L., Xu X., Zubielevitch E., Sibley C. G. (2023). Gain and loss spirals: Reciprocal relationships between resources and job insecurity. Journal of Occupational and Organizational Psychology.

[B28-behavsci-15-01593] Judd C. M., Kenny D. A. (1981). Process analysis: Estimating mediation in treatment evaluations. Evaluation Review.

[B29-behavsci-15-01593] Judd C. M., Kenny D. A., McClelland G. H. (2001). Estimating and testing mediation and moderation in within-subject designs. Psychological Methods.

[B30-behavsci-15-01593] Kiani F. S., Ehsan S. (2024). Association of positive psychological factors with the mental health of older adult retirees: A systematic review. International Journal of Human Rights in Healthcare.

[B31-behavsci-15-01593] Lai S., Lu L., Shen C., Yan A., Lei Y., Zhou Z., Wang Y. (2023). Income loss and subsequent poor psychological well-being among the Chinese population during the early COVID-19 pandemic. International Journal for Equity in Health.

[B32-behavsci-15-01593] Levasseur M., Généreux M., Bruneau J. F., Vanasse A., Chabot É., Beaulac C., Bédard M. M. (2015). Importance of proximity to resources, social support, transportation and neighborhood security for mobility and social participation in older adults: Results from a scoping study. BMC Public Health.

[B33-behavsci-15-01593] Liu C., Bai X., Knapp M. (2022). Multidimensional retirement planning behaviors, retirement confidence, and post-retirement health and well-being among Chinese older adults in Hong Kong. Applied Research in Quality of Life.

[B34-behavsci-15-01593] Liu X., Wang Z., Zhang C., Zhang C., Peng L., Xu H. (2024). Effects of income on subjective well-being in the elderly: Complete mediation roles of self-rated health and psychological capital. INQUIRY: The Journal of Health Care Organization, Provision, and Financing.

[B35-behavsci-15-01593] Locke E. A., Latham G. P. (1990). A theory of goal setting & task performance.

[B36-behavsci-15-01593] MacKinnon D. P., Sussman S. (2001). Commentary on Donaldson, mediator and moderator analysis in program development. Handbook of program development for health behavior research and practice.

[B37-behavsci-15-01593] Maslow A. H. (1943). A theory of human motivation. Psychological Review.

[B38-behavsci-15-01593] Moal G. M. (2021). The travel constraints faced by retired travelers in the 21st. Journal of Consumer Marketing.

[B39-behavsci-15-01593] Muratore A. M., Earl J. K. (2015). Improving retirement outcomes: The role of resources, pre-retirement planning and transition characteristics. Ageing & Society.

[B40-behavsci-15-01593] National Bureau of Statistics of China (2024). Statistical communiqué of the People’s Republic of China on the 2023 national economic and social development.

[B41-behavsci-15-01593] Noone J., O’Loughlin K., Kendig H. (2013). Australian baby boomers retiring ‘early’: Understanding the benefits of retirement preparation for involuntary and voluntary retirees. Journal of Aging Studies.

[B42-behavsci-15-01593] Noone J. H., Stephens C., Alpass F. (2009). Preretirement planning and well-being in later life: A prospective study. Research on Aging.

[B43-behavsci-15-01593] Noone J. H., Stephens C., Alpass F. (2010). The process of retirement planning scale (PRePS): Development and validation. Psychological Assessment.

[B44-behavsci-15-01593] Nunnally J. C. (1978). Psychometric theory.

[B45-behavsci-15-01593] Operario D., Adler N. E., Williams D. R. (2004). Subjective social status: Reliability and predictive utility for global health. Psychology & Health.

[B46-behavsci-15-01593] Palombi T., Chirico A., Cazzolli B., Zacchilli M., Alessandri G., Filosa L., Borghi A., Fini C., Antoniucci C., Pistella J., Alivernini F., Baiocco R., Lucidi F. (2025). Motivation, psychological needs and physical activity in older adults: A qualitative review. Age and Ageing.

[B47-behavsci-15-01593] Peeters M. C., Van Emmerik H. (2008). An introduction to the work and well-being of older workers: From managing threats to creating opportunities. Journal of Managerial Psychology.

[B48-behavsci-15-01593] Podsakoff P. M., MacKenzie S. B., Lee J. Y., Podsakoff N. P. (2003). Common method biases in behavioral research: A critical review of the literature and recommended remedies. Journal of Applied Psychology.

[B49-behavsci-15-01593] Preacher K. J., Rucker D. D., Hayes A. F. (2007). Addressing moderated mediation hypotheses: Theory, methods, and prescriptions. Multivariate Behavioral Research.

[B50-behavsci-15-01593] Reitzes D. C., Mutran E. J. (2004). The transition to retirement: Stages and factors that influence retirement adjustment. The International Journal of Aging and Human Development.

[B51-behavsci-15-01593] Roosevelt K. (2023). Adjusting to retirement: The effect of resources on psychological well-being from pre-to post-retirement. Ph.D. dissertation.

[B52-behavsci-15-01593] Rousseau D. (1995). Psychological contracts in organizations: Understanding written and unwritten agreements.

[B53-behavsci-15-01593] Rozynek C., Lanzendorf M. (2023). How does low income affect older people’s travel practices? Findings of a qualitative case study on the links between financial poverty, mobility and social participation. Travel Behaviour and Society.

[B54-behavsci-15-01593] Ryan R. M., Deci E. L. (2000). Self-determination theory and the facilitation of intrinsic motivation, social development, and well-being. American Psychologist.

[B55-behavsci-15-01593] Ryan R. M., Deci E. L. (2024). Self-determination theory. Encyclopedia of quality of life and well-being research.

[B56-behavsci-15-01593] Santini Z. I., Jose P. E., Cornwell E. Y., Koyanagi A., Nielsen L., Hinrichsen C., Meilstrup C., Madsen K. R., Koushede V. (2020). Social disconnectedness, perceived isolation, and symptoms of depression and anxiety among older Americans (NSHAP): A longitudinal mediation analysis. The Lancet Public Health.

[B57-behavsci-15-01593] Shang X. T., Wei Z. H. (2023). Socio-economic inequalities in health among older adults in China. Public Health.

[B58-behavsci-15-01593] Singh-Manoux A., Marmot M. G., Adler N. E. (2005). Does subjective social status predict health and change in health status better than objective status?. Psychosomatic Medicine.

[B59-behavsci-15-01593] Tang M., Wang D., Guerrien A. (2020). A systematic review and meta-analysis on basic psychological need satisfaction, motivation, and well-being in later life: Contributions of self-determination theory. PsyCh Journal.

[B60-behavsci-15-01593] Tang M., Wang D., Guerrien A. (2021). The contribution of basic psychological need satisfaction to psychological well-being via autonomous motivation among older adults: A cross-cultural study in China and France. Frontiers in Psychology.

[B61-behavsci-15-01593] Topa G., Moriano J. A., Depolo M., Alcover C. M., Morales J. F. (2009). Antecedents and consequences of retirement planning and decision-making: A meta-analysis and model. Journal of Vocational Behavior.

[B62-behavsci-15-01593] Wang M. (2007). Profiling retirees in the retirement transition and adjustment process: Examining the longitudinal change patterns of retirees’ psychological well-being. Journal of Applied Psychology.

[B63-behavsci-15-01593] Wang M., Henkens K., Van Solinge H. (2011). Retirement adjustment: A review of theoretical and empirical advancements. American Psychologist.

[B64-behavsci-15-01593] Wang Q., Timonen V. (2021). Retirement pathways and pension inequality in China: A grounded theory study. International Journal of Sociology and Social Policy.

[B65-behavsci-15-01593] World Health Organization (2021). Decade of healthy ageing: Baseline report.

[B66-behavsci-15-01593] Wu J., Chao Q. (2024). How older adults fulfill their retirement plans relates to positive mental health: A path model analysis of social activity and self-esteem. Current Psychology.

[B67-behavsci-15-01593] Yeung D. Y. (2013). Is pre-retirement planning always good? An exploratory study of retirement adjustment among Hong Kong Chinese retirees. Aging & Mental Health.

[B68-behavsci-15-01593] Yeung D. Y. (2018). Adjustment to retirement: Effects of resource change on physical and psychological well-being. European Journal of Ageing.

[B69-behavsci-15-01593] Yeung D. Y., Zhou X. (2017). Planning for retirement: Longitudinal effect on retirement resources and post-retirement well-being. Frontiers in Psychology.

[B70-behavsci-15-01593] Yuan J., Zhao B., Han B. (2025). The comprehensive impact of retirement planning achievement, active aging, and retirement adjustment on mental health: An empirical analysis. 2nd international conference on educational development and social sciences *(EDSS 2025)*.

[B71-behavsci-15-01593] Zell E., Strickhouser J. E., Krizan Z. (2018). Subjective social status and health: A meta-analysis of community and society ladders. Health Psychology.

[B72-behavsci-15-01593] Zhan Y., Froidevaux A., Li Y., Wang M., Shi J. (2023). Preretirement resources and postretirement life satisfaction change trajectory: Examining the mediating role of retiree experience during retirement transition phase. Journal of Applied Psychology.

[B73-behavsci-15-01593] Zhang J., Zhang Y., Shi Y., Han E. H. (2017). Reliability and validity of the Chinese version of the positive aging scale. Chinese Journal of Gerontology.

[B74-behavsci-15-01593] Zhang L. Y., Wang Z. J. (2019). Research status and localization development of retirement planning. Advances in Psychological Science.

[B75-behavsci-15-01593] Zhang M.-Y., Wang D.-H. (2018). Application of retirement adjustment questionnaire in Chinese retirees of state-owned enterprise. Chinese Journal of Clinical Psychology.

